# 非小细胞肺癌脑膜转移诊疗现状

**DOI:** 10.3779/j.issn.1009-3419.2015.10.05

**Published:** 2015-10-20

**Affiliations:** 100730 北京, 中国医学科学院北京协和医学院, 北京协和医院呼吸内科 Department of Respiratory Disease, Peking Union Medical College Hospital, Chinese Academy of Medical Science & Peking Union Medical College, Beijing 100730, China

**Keywords:** 肺肿瘤, 脑膜转移, 表皮生长因子受体酪氨酸激酶抑制剂, 靶向治疗, 鞘内注射化疗, Lung neoplasms, Leptomeningeal metastasis, EGFR-TKI, Targeted therapy, Intrathecal chemotherapy

## Abstract

脑膜转移(leptomeningeal metastasis, LM)是非小细胞肺癌(non-small cell lung cancer, NSCLC)的一个灾难性事件, 患者临床症状重, 预后极差。尽管鞘内注射化疗在晚期NSCLC的LM患者中显示一定的疗效, NSCLC-LM生存期仍仅为12周-14周。肺腺癌是NSCLC-LM患者主要的病理类型(84%-97%)。其中43.0%-70.5%的NSCLC-LM患者检测到表皮生长因子受体(epidermal growth factor receptor, *EGFR*)敏感突变。研究表明, 经选择的患者应用EGFR酪氨酸激酶抑制剂(tyrosine kinase inhibitors, TKIs)治疗LM有效, 可延长生存期。未来需要进一步的临床试验来验证EGFR-TKIs治疗的NSCLC-LM患者的疗效。

## Abstract (en)

Leptomeningeal metastasis (LM) is one of the disastrous events in managing advanced non-small cell lung cancer (NSCLC) due to severe clinical symptoms and a grave prognosis.Although intrathecal (IT) chemotherapy show some effects for LM in advanced NSCLC, the prognosis is still poor (12 wk-14 wk).A large majority (84%-97%) of the patients were found to have adenocarcinoma histology.Epidermal growth factor receptor (EGFR) senstive mutations were detected in 43.0%-70.5% adenocarcinoma patients with LM.EGFR tyrosine kinase inhibitors (TKIs) showed to be effective for LM in selected NSCLC patients in some reseaches, and confer a survival benefit.Furthermore, future trials need be done to determine the effect of EGFR-TKIs treatment in NSCLC-LM patients.

恶性肿瘤脑膜转移(leptomeningeal metastasis, LM)又称为脑膜癌病(meningeal carcinomatosis, MC), 是恶性肿瘤细胞在脑和脊髓的蛛网膜下腔内弥漫转移, 脑和脊髓的软脑(脊)膜弥漫性或多灶性、局限性肿瘤细胞浸润, 进而引起一系列的临床症状^[1]^。LM在恶性肿瘤中枢神经系统(central nervous system, CNS)转移发病率位于第3位, 且随着患者生存期的延长, LM发生率逐渐增高。随着靶向治疗的突破性的进展, 非小细胞肺癌(non-small cell lung cancer, NSCLC)患者生存期进一步延长, 中枢神经系统进展的发生率增高, 其中LM也是中枢神经肿瘤进展的一种重要表现^[2, 3]^。LM是NSCLC的一个灾难性的事件, 患者临床症状重, 如不进行治疗, 生存期仅4周-6周。因此, LM的治疗成为NSCLC治疗的一个难点。

## 1 流行病学

NSCLC易发生中枢神经系统转移, 30%-40%患者出现中枢神经系统转移, 其中约10%患者出现脑膜转移^[4, 5]^。确诊发生LM的NSCLC患者中^[6-11]^, 肺腺癌是最常见的病理类型, 约为84%-97%, 肺鳞癌仅占1%-6%, 肺腺癌的患者中, 约43.0%-70.5%为表皮生长因子受体(epidermal growth factor receptor, *EGFR*)敏感突变, 仅有个例的患者检测到*ALK*/*EML4*重排^[10]^。LM可发生在NSCLC治疗的任何阶段^[7, 9, 11, 12]^, 17.4%-22.0%患者诊断NSCLC的同时已发生LM, 其余患者在NSCLC化疗或靶向治疗的过程中出现LM, 此外, LM可作为NSCLC术后的唯一复发病灶, NSCLC发生LM的中位诊断时间为10个月-15个月。

## 2 病理生理

颅骨与脑实质间有三层膜, 由外向内为硬脑膜、蛛网膜和软脑膜。蛛网膜与软脑(脊)膜之间为蛛网膜下腔。脑脊液(cerebrospinal fluid, CSF)由第三脑室、第四脑室及侧脑室的脉络膜产生, 在脑室系统、蛛网膜下隙和脊髓中央管内循环, 通过上矢状窦的蛛网膜颗粒循环吸收入血。总量约140 mL, 每8 h循环一次。LM肿瘤转移的途径包括:血源转移到脉络膜丛血管/软脑膜血管/Batson静脉丛到达蛛网膜下腔, 沿神经或血管鞘进入蛛网膜下腔; 颅骨或脑实质的肿瘤转移病灶, 局部侵犯至肿瘤细胞进入蛛网膜下腔; 医源性治疗检查手段, 如脑肿瘤手术等可致肿瘤种植转移。进入蛛网膜下腔的瘤细胞, 通过CSF循环播散, 造成弥漫性或多灶性软脑膜浸润, 多发生于颅底、脊髓背侧及马尾。脉络膜、室管膜等部位形成的肿瘤结节样病灶影响CSF循环和吸收, 造成颅高压及脑积水, 可造成任何级别的神经轴的损坏; 肿瘤侵犯包绕神经的软脑膜可导致颅/脊神经根病变; 脊髓膜表面的肿瘤结节侵犯或压迫脊髓可导致脊髓相关症状^[1, 13]^。

## 3 临床表现

由于肿瘤细胞在蛛网膜下腔播散, 可到达神经系统的各个部位, 整个神经轴均可受累, 因此LM的临床症状复杂多样, 可表现为累及不同水平神经轴产生的多灶性症状和体征。肺癌患者如出现多样性和多发性的神经系统症状和体征, 需高度警惕LM, 但LM患者也可表现为孤立的神经系统受累表现, 如马尾综合征、单发颅神经受累表现。LM主要临床表现为以下三个方面^[13]^:①大脑半球脑膜受累症状:约发生于50%的患者, 表现为头痛、恶心、呕吐、头晕、行走困难、精神状态改变、意识丧失、认知障碍、感觉障碍、癫痫发作等; ②颅神经受累表现:约发生于40%患者, 动眼神经、滑车神经、外展神经受累可表现为眼肌麻痹, 进而引发复视, 听视神经受累可导致听力下降, 视神经受累至视野缺损, 面神经受累至周围性面瘫, 三叉神经、舌下神经、舌咽神经、迷走神经受累可至咀嚼吞咽障碍; ③脊髓和脊神经根受累表现:超过60%患者发生, 可表现为肢体无力、感觉异常、感觉性共济失调、膀胱和直肠括约肌功能障碍、神经根性疼痛, 查体发现颈项强直、腱反射减弱或消失、节段性感觉缺损、直腿抬高试验阳性等。此外, 约70%-80%NSCLC-LM患者同时合并脑转移^[6, 8, 9]^, 应注意与脑转移瘤相关症状相鉴别。

## 4 CSF检测

CSF检测包括CSF常规检测、CSF细胞学检测和CSF肿瘤标志物检测等。

### 4.1 常规检测

腰椎穿刺行CSF检查, 约40%-50%的患者CSF压力增高^[6, 11]^。此外, 检查可发现CSF细胞数增多, CSF蛋白增高, 葡萄糖降低, 氯化物降低。上述检测异常对于LM诊断有提示意义, 但缺乏特异性。.

### 4.2 细胞学检测

CSF找到肿瘤细胞是诊断LM的金标准。然而, 首次腰椎穿刺查CSF找肿瘤细胞的阳性率仅约50%, 2次CSF检测阳性率可升至80%, 而短期内进行3次以上的检查不提高检出阳性率^[14]^。CSF送检的随机性、CSF无法及时送检、CSF肿瘤细胞少、肿瘤细胞异型性少等原因, 可降低CSF的检出率。因此, 可通过以下措施提高检出率^[15]^:增加CSF送检标本量(超过10 mL), 标本及时送检, 如高度怀疑LM需重复送检, 以及请经验丰富的病理科医生病理读片等等。CSF细胞学免疫组化和CSF循环肿瘤细胞检测可一定程度地提高CSF肿瘤细胞^[16]^的检出率, 但目前NSCLC-LM相关研究仍很少。

### 4.3 CSF肿瘤标志物

CSF癌胚抗原(carcinoembryonic antigen, CEA)、神经原特异性烯醇化酶(neuron specific enolase, NSE)和细胞角蛋白19片断(cytokaratin 19 fragment, Cyfra21-1)升高对于LM有提示意义, 且LM患者CSF CEA、NSE及Cyfra21-1的浓度高于血浆浓度^[17]^。以CEA > 4.7 mμg/L、NSE > 14.6 mμg/L和CYFRA21-1 > 5.5 mμg/L作为CSF检测阳性判定标准, CEA或Cyfra21-1增高的敏感性为100.0%, 特异性91.4%, CEA和Cyfra21-1均增高的敏感性为74.3%, 而特异性高达100.0%。三者均阳性其诊断LM的特异性为100.0%, 三者之一阳性对于LM诊断的敏感性为100.0%。

### 4.4 CSF中*EGFR*基因突变检测

CSF标本可作为EGFR检测的一个标本来源, 无论是CSF中的游离DNA或是CSF肿瘤细胞, 均可检测*EGFR*突变。Yang等^[18]^应用ARMS法检测了30例肺腺癌中枢神经系统转移患者CSF的*EGFR*突变情况, 其中13例患者的CSF中检测到*EGFR*敏感突变。与组织中*EGFR*突变相比, 阳性预测值为75%(95%CI:0.45-1.00), 阴性预测值为75%(95%CI:0.51-0.99), 敏感性为67%(95%CI:0.36-0.97), 特异性为82%(95%CI:0.59-1.00)。其中7例患者合并LM, 5例患者检测到*EGFR*敏感突变。我院对于7例EGFR酪氨酸激酶抑制剂(tyrosine kinase inhibitors, TKIs)治疗过程中出现脑膜转移的患者进行CSF *EGFR*突变检测, 7例(100.0%)患者CSF均检测到*EGFR*基因突变。

## 5 影像学表现

磁共振成像(magnetic resonance imaging, MRI)对于LM诊断具有重要意义, LM患者的标准MRI评估需包括全脑及全脊髓增强MRI评估。LM增强MRI的典型表现包括^[13]^:①可延伸至脑沟回的线样或絮状强化影, 可为连续或局灶样分布, 也可表现为结节状, 此类的病灶多见于大脑半球的表面、大脑基底池、小脑幕和脑室室管膜表面; ②颅(脊)神级增强或增厚; ③硬膜下脊髓外位于脊髓蛛网膜腔内增强结节也多报道; ④另有8%-10%患者可发现脑室扩张等脑积水表现。对于合并典型临床症状的NSCLC患者, 增强MRI的典型表现可作为LM的一个诊断依据^[19, 20]^。可导致脑膜强化的疾病还包括急慢性脑膜炎、化学性脑膜炎及自身免疫病等, 需与LM相鉴别。

## 6 诊断

对于NSCLC患者, 如出现多样性和多发性的神经系统症状和体征, 或脑(实质)转移不能解释的神经系统症状体征, 需高度警惕LM。头痛、恶心、呕吐及脑膜刺激征是提示脑膜转移高颅压的典型临床表现, 但仍有很大一部分患者仅表现为颅(脊)神经损害或脊髓病变等神经系统症状体征, 对于此类患者应提高诊断意识。LM早期神经系统损伤小, 肿瘤负荷低, 患者一般情况好, 如能获得早期诊断和治疗, 可改善患者预后。依据2015年NCCN中枢神经系统肿瘤指南(NCCN Clinical Practice Guidelines in Oncology^TM^ Central Nervous System Cancer version 1, 2015), 对于NSCLC患者, 如有新发的提示LM的神经系统症状或体征, 需给予详细的神经系统查体评估, 如患者可得到进一步治疗需完善全脑全脊髓的增强MRI, 测定CSF压力、细胞数、葡萄糖、蛋白检测, 并完成细胞学病理诊断。如患者发现CSF肿瘤细胞阳性或影像学LM典型表现, 可诊断为NSCLC-LM。其中, CSF细胞学检测找到肿瘤细胞为诊断LM的金标准。然而, 仍有部分患者CSF细胞学和神经系统影像学表现为阴性, 仅有提示LM的异常CSF表现(高细胞数、蛋白增高、葡萄糖降低), 对于此类患者, 需重复腰椎穿刺复查CSF相关检查(图 1)。

**1:**
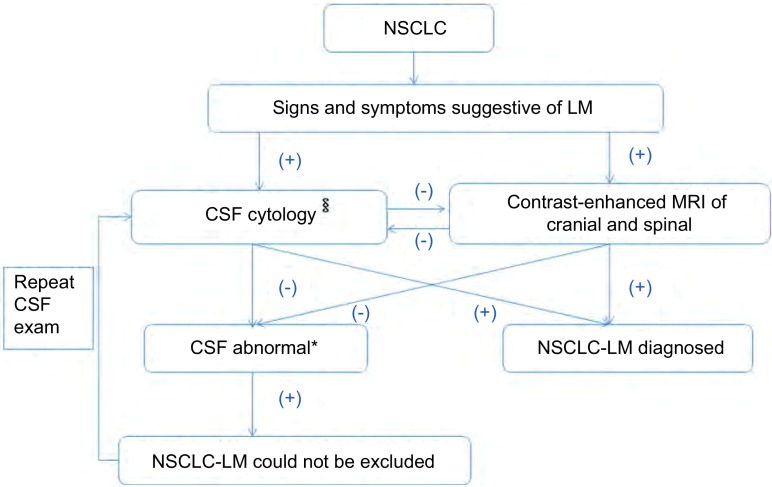
NSCLC-LM的诊断流程 Diagnostic approach to ascertain NSCLC-LM.^§^CSF analysis utilizes cytopathology to identify tumor cells.^*^:High WBC, low glucose and high protein.NSCLC:non-small cell lung cancer; LM:leptomeningeal metastasis; CNS:central nervous system; MRI:magnetic resonance imaging.

## 7 预后

如不进行积极治疗, LM患者的中位生存时间为4周-6周, 死亡原因多为LM相关的进行性的神经功能障碍和/或全身肿瘤进展^[1, 13]^。有效的治疗可以稳定或改善神经功能和症状, 提高患者生活治疗, 但即使接受传统治疗包括放疗、鞘内注射化疗等, 患者生存期仅至12周-14周^[9, 11]^。近年来, NSCLC治疗有了突破性的进展, 随着EGFR-TKIs的应用, NSCLC-LM患者的生存期较前延长, 将在下文中进一步阐述。目前研究^[6, 8, 11]^发现, 提示LM预后好的指标包括:肺腺癌、存在*EGFR*突变、接受系统性治疗(全身化疗或EGFR-TKIs)、鞘内注射化疗(intrathecal chemotherapy, ITC)、脑室腹腔分流(ventriculo peritoneal shunt), 对于治疗有反应; 提示LM治疗效果差的指标包括:年龄≥60岁、体力评分差[卡氏体力状态(Karnofsky performance status, KPS) < 70分或东部肿瘤合作组(Eastern Cooperative Oncology Group, EOCG) > 2分]、CSF细胞数高(WBC > 7/mm^3^)、CSF蛋白高(> 40 mg/dL)、不可控制的颅高压、多发神经系统损害及LM相关脑病(急性或亚急性的精神混乱综合征)等。LM治疗效果差的原因包括:①血脑屏障使得系统性治疗药物难以通过血脑屏障达到有效治疗浓度; ②肿瘤细胞在CSF总广泛播散, CSF循环受阻, 使得鞘内注射化疗药物难以到达肿瘤病灶区域; ③鞘内注射可选的药物如甲氨蝶呤和阿糖胞苷对于NSCLC敏感度差; ④患者多为肿瘤晚期, 一般情况差, 难以耐受有效的治疗, 如全脑全脊髓放疗、系统性化疗等; ⑤晚期的NSCLC患者CSF肿瘤细胞可能为耐药细胞, 对治疗效果差。

## 8 治疗

因为肿瘤细胞可经过CSF循环在蛛网膜下腔广泛播散, 脑膜转移的治疗应包括整个神经轴。目前针对NSCLC-LM的治疗手段包括支持治疗、外科治疗、放疗、鞘内注射化疗、全身化疗、靶向治疗等。

### 8.1 支持治疗

对于颅高压的患者需积极给予脱水降颅压治疗, 可选药物包括:甘露醇、甘油果糖和呋塞米。糖皮质激素可减轻脑水肿, 对于重度颅高压患者可应用, 但不改善预后。对于有癫痫发作的患者, 需加用抗癫痫治疗。疼痛明显患者可给予对症止痛治疗。尽管支持治疗可一定程度上缓解症状, 但如无法进行针对LM的治疗, 单纯支持治疗效果会随着时间的延长效果逐渐减弱。

### 8.2 手术治疗

由于CSF肿瘤细胞在CSF中循环播散, 手术治疗无法达到消除肿瘤的目的, 手术治疗主要作为一种辅助治疗措施, 目前采取的主要策略为手术行Ommaya储液囊植入或VP分流。通过立体定向引导下行侧脑室穿刺并植入Ommaya储液囊, 经Ommaya储液囊行脑室内化疗, 可减少反复行腰椎穿刺而加重的患者心理负担。105例接受脑室内化疗NSCLC-LM患者进行了回顾性分析^[6]^, 59例患者接受了Ommaya储液囊, 46例患者接受了化疗泵(置入方式类似于Ommaya储液囊, 但在颅骨表面进行一定程度的雕刻, 使得化疗泵嵌入于颅骨表面)。这些患者给予规律的脑室内注射化疗, 对于颅内高压的患者抽取多至30 mL的CSF以达到缓解颅内高压的目的。该组患者的中位生存期为3(范围:0.5-21.5)个月。对于颅内高压脑积水患者行脑室-腹腔分流, VP分流可显著降低患者颅内高压, 可显著缓解颅内高压引起的脑积水, 改善患者颅内高压引发的相关脑病, 患者一般情况(KPS评分), 且VP分流可延长患者生存期^[11, 21]^。Lee等^[11]^回顾性分析了149例NSCLC-LM患者的临床资料, 其中23例患者完成了VP分流, 多因素分析显示, VP分流是预后好的因素之一(*P*=0.013)。多项案例报道^[22, 23]^也提示, 对于NSCLC患者行VP分流联合EGFR-TKIs及鞘内注射化疗治疗, 可显著延长患者的生存期(5个月-15个月)。

### 8.3 放疗

放疗是脑转移癌的一种重要治疗手段, 但是对于LM患者, 疗效是不确定的。由于肿瘤细胞在CSF广泛播散, LM患者需接受全脑全脊髓放疗。由于全脑全脊髓放疗毒性大, 且患者一般情况差, 患者无法耐受, 治疗相关副作用大, 不延长生存期。部分LM患者可接受全脑放疗(whole brain radiotherapy, WBRT), 全脑放疗多采取为30 Gy/10 f的治疗模式, 或是针对局部病变进行放疗。部分研究认为WBRT并不能改善预后。实体瘤脑膜转移患者(乳腺癌及肺癌), 如仅接受全脑放疗, 其生存期仅8.1周^[24]^。Morris等^[9]^回顾性分析了125例NSCLC-LM患者, 其中46例接受全脑放疗而59例未接受全脑放疗, 两组患者生存期无显著差异。Umemura等^[8]^回顾分析了91例NSCLC-LM患者资料, 其中21例患者(23%)接受放疗, 研究结果显示放疗不改善预后。然而, 也有研究提示WBRT可能一定程度地改善预后。Lee等^[11]^的研究中, 149例患者中, 65例患者接受放疗联合其他治疗, 多因素分析显示, WBRT是预后好的指标之一(*P*=0.009)。放疗对于局灶性病灶的消除从而降低CSF中瘤负荷、改善血脑屏障通透性使得系统性治疗药物CSF浓度增高以及放疗对于脑实质内肿瘤病灶的治疗, 可能是其改善预后的原因。值得注意的是, 由于放疗可导致一过性的脑水肿, WBRT需在LM患者颅压控制的前提下进行。综上, 全脑放疗对于NSCLC-LM患者疗效不确定, 多数研究认为单纯全脑放疗在总生存期上无获益, 但是有研究显示全脑放疗联合系统治疗可一定程度改善预后。因此, 全脑放疗联合化疗或者分子靶向治疗有可能在一定程度上改善NSCLC-LM患者的预后, 值得临床进一步研究。

### 8.4 鞘内注射化疗

鞘内注射化疗是LM治疗的主要手段之一, 将药物直接注入蛛网膜下腔, 使得CSF中达到一定的药物浓度, 从而杀伤肿瘤细胞。目前可采取鞘内注射治疗的途径包括:经腰椎穿刺将药物打入蛛网膜下腔, 或行Ommaya储液囊植入术经Ommaya储液囊行脑室内化疗。由于化疗药物的毒性因素, 仅有少数药物可用于鞘内注射化疗, 如甲氨蝶呤(methotrexate, MTX)、阿糖胞苷(Cytarabine, Ara-C)、噻哌啶等, 但此类药物鞘内注射用药多为参考血液肿瘤脑膜转移治疗方案, 对肺癌细胞仅中度敏感。鞘注化疗药物同时给予鞘注糖皮质激素, 可减轻化疗药物对神经系统的毒性作用; 并抑制肿瘤细胞对中枢神经系统的毒性作用, 暂时改善并缓解临床症状。目前NSCLC-LM可选的方案^[1]^包括:①MTX:诱导治疗为MTX 10 mg-15 mg, 每周2次, 共4周, 此后每周1次, 共4周, 行巩固治疗, 再给予每月1次维持治疗; ②Ara-C:Ara-C每次25 mg-100 mg, 每周2次, 共4周, 行诱导治疗, 此后每周1次, 共4周, 行巩固治疗, 再给予每月1次的维持治疗; ③脂质体阿糖胞苷(Dep℃yt)^[25]^:Dep℃yt 50 mg每2周1次, 共8周为诱导化疗, 此后50 mg每4周1次, 共24周, 为维持治疗; ④三药联合方案:MTX 15 mg+Ara-C 30 mg/m^2^+氢化可的松15 mg/m^2^, 每周2次, 至CSF肿瘤细胞转阴后每周1次维持^[26]^。行鞘内注射化疗可改善NSCLC-LM患者预后, 但不同药物对于患者生存期的影响差异不明显^[1]^。Lee等^[11]^的回顾性研究中, 109例(73.2%)患者接受鞘内注射化疗(MTX 15 mg, 每周2次), 接受鞘内注射化疗的中位次数为9次(范围:1-27), 仅15例患者实现细胞学转阴, 研究提示鞘内注射化疗是改善预后的重要指标(*P* < 0.001)。Gwak等^[6]^分析了105例患者, 行脑室内注射化疗, 40例接受MTX单药化疗(MTX 15 mg), 65例接受了三药联合化疗[MTX 15 mg+Ara-C 30 mg/m^2^+氢化可的松(hydrocortisone)15 mg/m^2^], 上述方案每周2次, 直至CSF转阴, 或完成VP分流, 或患者拒绝。平均每例患者完成了5次脑室内注射化疗。经鞘内注射化疗后, 经过鞘内注射化疗8例患者细胞学转阴, 42%患者头痛症状改善, 仅18%(7/38)患者精神心理状态好转, 仅15%(2/13)例患者马尾综合征症状缓解, 13%(2/16)患者颅神经症状改善。该组患者治疗过程中有29例患者新发精神心理状态改变, 14例患者新发马尾综合征, 4例患者出现颅神经病变。该组患者中位生存期为3个月。

### 8.5 全身化疗

NSCLC系统性化疗有效的药物, 血脑屏障的通透性差, 且NSCLC-LM患者一般情况差, 通常难以耐受标准化疗方案。然而, 由于LM患者的血脑屏障受一定程度的破坏, 如患者可耐受的情况下, 全身化疗可改善患者预后。目前关于NSCLC-LM可选的化疗药物包括培美曲塞、长春瑞滨、吉西他滨、多西他赛、顺铂、替莫唑胺等。Gwak等^[6]^的研究中, 24例患者鞘注的同时或序贯给予全身化疗, 多因素分析提示全身化疗可改善患者预后。Park等^[7]^报道了8例接受全身化疗的患者, 分别应用多西他赛、长春瑞滨、吉西他滨联合顺铂以及培美曲塞的治疗, 患者生存期延长。替莫唑胺血脑屏障通过率高, 对于脑胶质瘤相关的脑膜转移有一定效果, 但一项Ⅱ期临床研究^[27]^发现, 对于肺癌及乳腺癌的脑膜转移患者临床获益率仍很低[3/19(15.8%)], 其中位生存期仅43天。有研究发现, 应用培美曲塞联合厄洛替尼^[28]^或吉非替尼^[29]^, 可能对于EGFR-TKIs治疗过程中发生脑膜转移的患者有效。

### 8.6 靶向治疗

#### 8.6.1 EGFR-TKIs治疗

对于存在EGFR敏感的NSCLC患者, EGFR-TKIs靶向治疗可显著延长患者生存期。通过本文对于NSCLC-LM流行病学总结发现, NSCLC-LM患者以腺癌为主要病理类型(84%-97%), *EGFR*突变发生率高(43.0%-70.5%)。EGFR-TKIs可用于具有敏感突变的NSCLC-LM患者。由于EGFR-TKIs毒副作用小, 即使患者ECOG评分差也可接受治疗。

EGFR-TKIs是小分子的靶向治疗药物, 能一定比例透过血脑屏障。我中心关于肺腺癌患者CSF吉非替尼药物浓度监测的研究^[30]^发现, CSF与血浆吉非替尼浓度比为(1.3%±0.7%)。且脑转移可导致CSF吉非替尼浓度增高。另有研究^[31-33]^表明, 厄洛替尼也可一定程度地透过血脑屏障, CSF与血浆浓度比约为4%-7%。因此EGFR-TKIs对于NSCLC的原发灶、脑转移灶乃至LM都有治疗作用。

EGFR-TKIs治疗可显著延长NSCLC-LM患者生存期。Yi等^[34]^回顾了11例*EGFR*敏感突变或是高度提示*EGFR*突变的LM患者, 其中9例患者接受厄洛替尼150 mg, 每天1次, 2例患者大剂量吉非替尼(500 mg/d和750 mg/d)继以厄洛替尼治疗, 其中6例患者为应用吉非替尼的过程中出现病情进展。9例患者同时给予MTX鞘内注射, 4例患者接受全脑放疗。经治疗后9例患者临床症状改善。该组患者的生存期为2.5个月-18.6个月, 中位总生存期(overall survival, OS)未达。Park等^[7]^回顾了50例细胞学确诊的NSCLC-LM患者, 其中14例患者接受了EGFR-TKIs治疗患者, 其中位OS达19.2个月, 生存期明显延长。

*EGFR*突变情况对于NSCLC-LM治疗具有重要意义。Umemura等^[8]^回顾了91例NSCLC-LM临床资料, 51例患者接受EGFR-TKIs治疗, 接受EGFR-TKIs治疗的患者较未接受EGFR-TKIs治疗患者生存期明显延长(5.3个月 *vs* 2.3个月, *P* < 0.001)。其中30例行*EGFR*突变检测, 结果显示, 7例患者为野生型, 10例患者为21外显子点突变, 13例患者为19外显子缺失突变。经EGFR-TKIs治疗, 敏感突变患者一般情况明显好转, 而无突变的患者情况无改善。野生型患者OS为1.4个月, 有趣的是, 而21外显子点突变患者OS为7.1个月, 而19缺失突变患者的生存期可达11个月(*P* < 0.001)。研究结果显示*EGFR* 19外显子缺失突变NSCLC-LM生存期更长。

靶向治疗药物对于NSCLC-LM治疗有一定影响。Lee等^[35]^对25例接受EGFR-TKIs的患者进行了回顾性研究。其中9例有21外显子点突变, 8例为19外显子缺失突变, 野生型3例, 不详5例, 9例此前接受EGFR-TKIs治疗。最终14例患者接受厄洛替尼治疗, 11例患者接受吉非替尼治疗, 所有患者均行鞘内注射化疗, 4例患者接受WBRT。结果显示厄洛替尼组患者CSF细胞学转阴率高(64.3% *vs* 9.1%, *P*=0.012)。厄洛替尼组OS为9.5个月, 吉非替尼组患者OS为4.4个月, 由于样本量小, 二者无统计学差异(*P*=0.960), 需进一步大样本临床试验进行验证吉非替尼与厄洛替尼在治疗LM中的疗效差异。由于厄洛替尼的CSF通过率高, 在CSF中可达到有效抗肿瘤浓度^[31-33]^, 吉非替尼治疗过程中发生LM可改用厄洛替尼治疗^[22]^。

综合治疗也至关重要。由于EGFR-TKIs毒副作用小, 患者可耐受靶向治疗的同时进行综合治疗。在上述研究中, 接受EGFR-TKIs治疗的患者同时也接受了鞘内注射化疗, 部分患者行全脑放疗或全身化疗, 另有极少的患者行VP分流。以靶向治疗为基础的综合治疗显著延长患者的生存期。

#### 8.6.2 间变淋巴瘤激酶(anaplastic lymphoma receptor tyrosine kinase, ALK)抑制剂治疗

ALK重排阳性的NSCLC可应用ALK-TKIs治疗, 克唑替尼是第一个上市的ALK-TKI药物。由于ALK阳性NSCLC发生率低, *ALK*重排的NACLC-LM更为罕见^[10]^, 仅有个例报道^[36-38]^。未接受克唑替尼治疗的LM患者给予克唑替尼效果好, 克唑替尼治疗过程中发生LM, 治疗效果差。

#### 8.6.3 贝伐珠单抗(Bevacizumab)

LM患者CSF的表皮生长因子受体(vascular endothial growth factor, VEGF)增高, 因此, 贝伐珠单抗可能对于LM有效。有案例^[39]^报道全身应用贝伐珠单抗联合化疗对于乳腺癌脑膜转移有一定疗效, 但对于NSCLC, 目前尚无相关报道, 需进一步研究和探讨。鞘内注射贝伐珠单抗目前尚处于动物研究阶段。

## 9 结论

LM是NSCLC的一个灾难性事件, 如不进行治疗, 患者预后极差。LM的临床症状复杂多样, 可表现为累及不同水平神经轴产生的多灶性症状和体征。对于怀疑LM的患者需行腰椎穿刺进行CSF细胞学检测并完善神经系统MRI, 以尽快明确诊断并进行积极治疗。鞘内注射化疗、全脑放疗、全身化疗以及VP分流是NSCLC-LM治疗的可选择的治疗措施。NSCLC-LM中腺癌是一个重要病理类型, NSCLC-LM患者*EGFR*突变发生率高。对于存在*EGFR*敏感突变的患者, EGFR-TKIs为基础的综合治疗可改善患者症状, 显著延长患者生存期。新的治疗模式需进一步大样本的临床分析进行验证。
